# The specificity of attentional biases by type of gambling: An eye-tracking study

**DOI:** 10.1371/journal.pone.0190614

**Published:** 2018-01-31

**Authors:** Daniel S. McGrath, Amadeus Meitner, Christopher R. Sears

**Affiliations:** Department of Psychology, University of Calgary, Calgary, Alberta, Canada; Ariel University, ISRAEL

## Abstract

A growing body of research indicates that gamblers develop an attentional bias for gambling-related stimuli. Compared to research on substance use, however, few studies have examined attentional biases in gamblers using eye-gaze tracking, which has many advantages over other measures of attention. In addition, previous studies of attentional biases in gamblers have not directly matched type of gambler with personally-relevant gambling cues. The present study investigated the specificity of attentional biases for individual types of gambling using an eye-gaze tracking paradigm. Three groups of participants (poker players, video lottery terminal/slot machine players, and non-gambling controls) took part in one test session in which they viewed 25 sets of four images (poker, VLTs/slot machines, bingo, and board games). Participants' eye fixations were recorded throughout each 8-second presentation of the four images. The results indicated that, as predicted, the two gambling groups preferentially attended to their primary form of gambling, whereas control participants attended to board games more than gambling images. The findings have clinical implications for the treatment of individuals with gambling disorder. Understanding the importance of personally-salient gambling cues will inform the development of effective attentional bias modification treatments for problem gamblers.

## Introduction

Pathological gambling was reclassified as a behavioral addiction in the latest edition of the Diagnostic and Statistical Manual of Mental Disorders [1]. The newly termed *gambling disorder* is a psychological condition that often results in severe psychosocial and financial distress [2–3]. Contemporary models of gambling disorder propose that a number of underlying mechanisms can influence the maintenance of gambling behaviour, including attentional biases. In the addictions literature, an attentional bias is said to occur when an individual allocates disproportionally more attention to addiction-relevant cues than to neutral stimuli [4–5]. A growing body of evidence has documented that disordered gamblers often exhibit disproportionally more attention to gambling-related stimuli relative to non-gambling stimuli [6].

In their influential incentive sensitization theory of addiction, Robinson and Berridge [7] proposed that environmental cues repeatedly paired with substance use acquire incentive salience through associative learning mechanisms. Specifically, drug use activates the mesocortico-limbic dopamine system and results in sensitization of the brain’s reward circuitry following consistent use. Once sensitized, the individual experiences enhanced psychological ‘wanting’ of the substance and is highly susceptible to cue-induced craving upon exposure to drug-related stimuli [8–9]. While primarily tested in substance use populations (i.e., ingested substances), theorists have proposed that a similar process also occurs within behavioral addictions such as disordered gambling [10–12].

### Attentional biases in disordered gamblers

In their systematic review, Hønsi et al. [6] identified 11 studies that reported that disordered gamblers exhibited an attentional bias for gambling-related stimuli in laboratory attention tasks. These studies used a variety of different tests for assessing attentional bias, including the Stroop task, dot-probe tasks, the attentional blink task, lexical salience tasks, and flicker-induced change blindness tests. Although results were mixed, overall, most studies reported evidence of an attentional bias in disordered gamblers (e.g., [13–17]), whereas four studies did not [18–21]. Most recently, two experiments that used a modified version of the Posner cueing task found evidence of attentional biases in disordered gamblers. In the first, abstinent gamblers in treatment were found to have an avoidance bias away from gambling stimuli, whereas disordered gamblers not in treatment displayed preferential attention toward gambling images (e.g., slots, chips, lottery tickets) over neutral pictures (e.g., petrol pumps, buttons, watches) [22]. In the second experiment, non-disordered and disordered gamblers completed a Posner task in which gambling stimuli presentation times were manipulated to allow for separate assessments of the initial orientation of attention and the maintenance of attention [23]. Disordered gamblers displayed a bias toward gambling stimuli during the initial orientation of attention, whereas non-disordered gamblers did not. Furthermore, it was also found that disordered gamblers experienced higher gambling craving and negative mood states, factors postulated to influence the attentional bias in this group. Taken together, the literature supports the existence of an attentional bias for gambling stimuli among disordered gamblers.

### Eye-tracking studies of attentional biases in gambling

Although several different tasks have been used to examine attentional biases in gamblers, the potential of eye-tracking technology has been largely overlooked. Eye-gaze tracking provides a direct and continuous measure of a participant’s attention by recording individual fixations to stimuli as they are attended to. Moreover, eye-gaze tracking has been shown to display superior reliability when compared to other measures of attentional bias [24]. Despite these advantages, only two studies have used this methodology to examine attentional biases in gamblers. Brevers et al. [25] combined eye-gaze tracking with a change detection task. Pairs of gambling and neutral images were displayed for 250 ms and separated by a mask presented for 80 ms. Disordered gamblers exhibited a significantly greater attentional bias toward gambling-related images compared to controls. Most recently, Grant and Bowling [26] combined eye-tracking with a dot-probe task, presenting pairs of gambling and neutral images for 500 ms and 2000 ms to a sample of non-disordered gamblers. As predicted, positive attitudes toward gambling, as well as gambling frequency, were significantly correlated with fixation times to gambling images. On the other hand, contrary to expectations, the proportion of initial eye-movements toward gambling images was not. Although neither of these studies was a pure eye-tracking task (in both cases eye-tracking was paired with a task that required a speeded manual response), the results of these studies indicate that eye-gaze tracking is an effective tool for measuring attentional biases for gambling stimuli.

### Heterogeneity by preferred type of gambling

One consideration that appears to have been overlooked in the attentional bias literature is the degree of heterogeneity in gambling behavior among disordered gamblers. Indeed, gamblers are a heterogeneous group and important differences exist between individuals who gravitate toward specific forms of gambling versus others. Disordered gamblers presenting for treatment have been found to differ according to gambling severity, co-morbid addictions, and other psychiatric diagnoses based on the type of gambling activity they prefer [27]. Moragas et al. [28] provided evidence for distinct phenotypes of gamblers based on preferred gambling activity. For example, disordered gamblers who prefer strategic gambling are more likely to be male, engage in more gambling activities, spend more money gambling, and present with other forms of psychopathology. In contrast, non-strategic gamblers are more likely to be female and more likely to gamble as a means of regulating negative emotions. Other research suggests that strategic gamblers (e.g., cards, dice, sports betting, stock market) and non-strategic gamblers (e.g., electronic gaming, pull tabs) differ from controls on measures of cognitive inflexibility and impulsivity, but not from each other (e.g., [29]).

Despite these important differences, most studies of attentional biases in gambling have not taken the heterogeneity of gambling behavior into account in their participant recruitment or in their choice of experimental stimuli. For example, Brevers et al. [25] and Grant and Bowling [26] selected participants primarily using gambling severity scores on the South Oaks Gambling Screen (SOGS) [30] and the Problem Gambling Severity Index (PGSI) [31], respectively. Limited information was provided on the gambling behaviours of the participants, and the types of gambling activities participated in, the intensity of gambling participation, and overall gambling preferences of participants were not described. The experimental stimuli included several types of gambling imagery (e.g., poker, roulette, dice, sports, horse racing, lotteries) and were not specific to any one type of gambling. While such stimuli are clearly “gambling-related”, it is unknown if each (or any) gambling activity had direct personal relevance for individual participants. For example, it is possible that a participant may identify as a poker player, but have little or no experience with other forms of gambling such as horse racing or roulette. Labelling participants as “gamblers” and categorizing stimuli as “gambling-related” likely limits researchers’ ability to identify potential specificities in attentional biases among different types of gamblers.

### Rationale

The purpose of this study was to determine the extent to which attentional biases in disordered gamblers are associated with preferred gambling activities. Specifically, the degree of alignment between preferred gambling and attentional bias for congruent gambling imagery was investigated using eye-gaze tracking to measure attention. Individuals who were primarily ‘poker players’, ‘video lottery terminal (VLT)/slot machine players’, and non-gambling controls were recruited from the local community for a single test session. Poker and VLT/slot gambling were chosen because they are commonly endorsed forms of gambling in students and the general population [32–33] and are highly representative of ‘strategic’ and ‘non-strategic’ gambling, respectively [34–35]. The present study also differs from previous eye-gaze tracking studies in the way that gambling- and non-gambling-related stimuli were presented: images were presented over a much longer interval (8 seconds) and eye-tracking data was collected throughout this 8-sec interval, which provided a more nuanced measure of gambling-related attentional bias. The existence of an attentional bias in disordered gamblers under these conditions, which more closely resemble natural viewing conditions, would document the generalizability of this phenomenon beyond response latency-based attention tasks.

It was hypothesized that both gambling groups would preferentially attend to images associated with their gambling activity of choice relative to non-preferred gambling images (i.e., bingo, and poker or VLT/slots, depending on the type of gambler) and relative to non-gambling control images (board games). That is, poker players were predicted to preferentially attend to poker images and VLT players were predicted to preferentially attend to VLT images. It was also predicted that non-gamblers would attend equally to gambling and non-gambling control images due to an absence of a gambling-related attentional bias.

## Material and methods

### Participants

A total of 80 male participants (31 poker players, 19 VLT/slots players, and 30 non-gambling controls) were recruited from two sources: undergraduate students recruited from an online research participation system, and members of the local community recruited through advertisements placed on Internet bulletin boards and in newspapers. All participants were males between 18 and 35 years old (*M* = 21.9 years, *SD* = 3.6). Participation was restricted to younger males in order to reduce potential extraneous effects of sex and age on the results. The two gambling groups were defined using the following criteria: 1) for pokers players, individuals who had spent money playing poker in the past 3 months, had not gambled on VLTs in the past 3 months, and reported poker as the gambling activity that they had spent the most money on, 2) for VLT players, individuals who had spent money playing VLTs in the past 3 months, had not gambled on poker in the past 3 months, and reported VLTs as the gambling activity that they had spent the most money on. Non-gambling controls were required to score 0 on the PGSI [31] and to have not gambled for money on most gambling activities in the previous 12 months (lottery and raffle tickets being the exceptions). Exclusion criteria for all groups included color blindness (which could affect how images are viewed), current treatment or past seeking of treatment for a gambling-related problem, and intention to quit gambling in the next 30 days.

### Ethics statement

This experiment was conducted in accordance with the ethical principles outlined in the Declaration of Helsinki. Prior to testing, all measures and procedures underwent ethical review by the Conjoint Faculties Research Ethics Board (REB15-2828) at the University of Calgary.

### Demographics and gambling involvement

All demographic information (e.g., age, sex, ethnicity, marital status, education) was collected using a self-report questionnaire. The questionnaire also included items on gambling history and substance use.

### Gambling severity

Gambling severity was measured with the Problem Gambling Severity Index (PGSI) from the Canadian Problem Gambling Index (CPGI) [31]. The PGSI contains 9 items, each rated on a scale from 0 (*never*) to 3 (*almost always*). Items include: “Have you felt that you might have a problem with gambling?” and “When you gambled, did you go back another day to try to win back the money you lost?”. Higher total scores indicate greater gambling severity, with scores of 5 or greater indicating probable disordered gambling [36]. The PGSI has been shown to have excellent psychometric properties [37].

### Apparatus

#### Eye-gaze tracking

Eye-tracking data was collected using an Eyelink 1000 eye-gaze tracking system (SR Research Ltd., Ottawa, Ontario), which measures eye movements using an infrared camera. The sampling rate of the system is 1000 Hz, with an average gaze error of less than 0.5 degrees of visual angle. Stimuli were presented on a 24-inch LCD monitor positioned approximately 60 cm away from the participant.

#### Image stimuli

**Fig 1:**
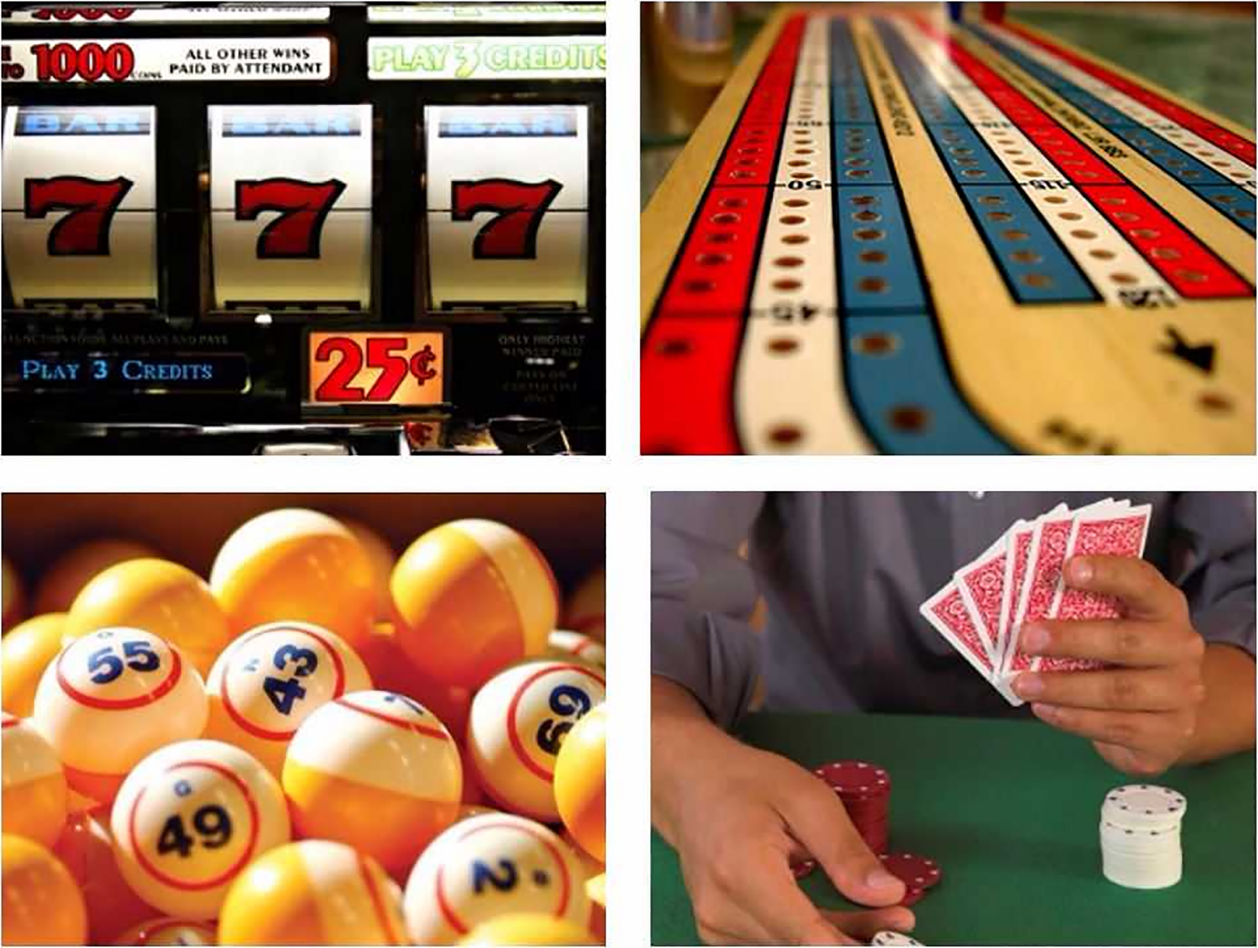
Sample experimental trial. Sample experimental trial containing 4 images (bingo, poker, VLT/slots, board games). Each trial was displayed for 8 seconds and contained novel images for each category (i.e., each image shown only once). There were a total of 25 experimental trials plus 2 practice trials.

The experimental stimuli consisted of 108 high-resolution color images presented across 25 trials (plus 2 practice trials, for a total of 27 trials). Each image was 300 hundred pixels in height x 400 hundred pixels in width. The video display had a resolution of 1024 × 768 pixels and the images were shown on a white background. As noted, each trial consisted of four images: one poker image, one VLT/slot machine image, one bingo image, and one board game image (see Fig 1). Images were carefully chosen to be representative of each category and to be devoid of other addictive substances (e.g., tobacco, alcohol, illicit drugs). Bingo images were chosen to assess the extent to which poker and VLT gamblers would attend to a different form of gambling. Board games served as a non-gambling neutral category (e.g., chess, checkers, crib, Scrabble, Monopoly, etc.). Each image was unique and shown only once in the 27 trials.

### Procedure

Prospective participants first completed a telephone screening procedure to assess their eligibility for inclusion in the study. Only participants meeting eligibility criteria were invited to the laboratory. During the laboratory visit, participants first completed an informed consent procedure in a room that contained no gambling-related cues. Next, they were escorted by a research assistant to a separate dedicated eye-tracking room and seated at the eye-tracker. Participants were provided with written and verbal instructions prior to testing. Specifically, they were asked to view a slideshow of images and to look at the images in any way they desired. They were told that the movement of their eyes would be tracked by the eye-tracking system. Participants used a chinrest to reduce head movements during data collection, in order to increase tracking accuracy. Prior to data collection, a calibration procedure was completed for each participant to ensure accurate gaze measurement (this took approximately 5 minutes). Following the calibration, the presentation of the 25 sets of images commenced. Each trial began with the presentation of a fixation marker (a black circle with a white dot in the middle) in the center of the display for 3 seconds. Four images were displayed during each 8-second presentation (bingo, VLT/slots, poker, and board game images, one from each category), and every presentation included an image from each of these categories. The 8-second presentation time was chosen to ensure that a clear measure of sustained attention to the images would be obtained; this presentation time has been used successfully in previous studies employing similar designs (e.g., [38–39]). The four images were located in the four quadrants of the screen (top left, top right, bottom left, bottom right quadrants). The image categories were randomized by the eye-tracking software such that each image could randomly appear in any quadrant during any trial. The order of trials was randomized separately for each participant and the data was collected in approximately 5 minutes. After the eye-tracking data was collected, participants returned to the test room and completed questionnaires. Finally, participants were debriefed as to the purpose of the study and compensated with a gift card valued at $20 CAD or bonus course credit (a 1% increase in the final grade of a psychology course of their choosing). The total time to complete the study was between 30 and 45 minutes.

### Data analysis

Eye-gaze tracking data was first screened for recording artifacts, eye-blinks, and missing data using the EyeLink Data Viewer analysis software (SR Research), using the standard settings. For the statistical analyses, there were four dependent variables of interest: first fixations, time of first fixation, first dwell time, and total fixation time (all measured in milliseconds). The first fixation variable is defined as the first image fixated on in each trial. Time of first fixation is defined as the earliest point in a trial that an image is fixated on, for any duration; images that capture participant’s attention have shorter first fixation times. First dwell time is defined as the amount of time that an image is fixated on immediately following its first fixation. Longer first dwell times reflect greater interest in an image the first time it is attended to. Total fixation time is the total amount of time an individual fixates on an image during the 8-second presentation. Longer total fixation times reflect greater interest and attentional engagement with an image. Each of these dependent variables were analyzed separately, using a 3 (Group: controls, poker players, VLT) x 4 (Image Type: poker, VLT/slots, bingo, board games) mixed-model analyses of variance (ANOVA). Correlations between PGSI severity scores and total fixation time among poker players and VLT players were also examined to assess the relationship between gambling severity and attentional bias for gambling imagery.

## Results and discussion

### Participant characteristics

Table 1 shows demographic comparisons between the three groups. An analysis of variance (ANOVA) indicated that there was a significant difference between the groups on age, *F*(2, 77) = 5.12, *p* = .01, partial η^2^ = .12. Pairwise comparisons revealed that poker players (*M =* 23.4, *SD =* 4.2) were significantly older than VLT players (*M =* 21.3, *SD =* 2.5), *p* = .04, and controls (*M =* 20.7, *SD =* 2.9), *p* = .01, whereas VLT players did not differ from controls, *p* = .56. Note that the age differences were quite small, with the largest difference (between poker players and controls) being an average of 2.7 years.

**Table 1:** Demographic characteristics of the three participant groups.

	Poker Players (*n* = 31)	VLT Players (*n* = 19)	Controls (*n* = 30)
	*% / M(SD)*	*% / M(SD)*	*% / M(SD)*
Age	23.4 (4.2)	21.3 (2.5)	20.7 (2.9)
Marital status			
Single	90.3%	83.3%	93.3%
Married/cohabitating	9.7%	16.7%	6.7%
Ethnicity			
Caucasian	51.6%	52.6%	30.0%
Asian	32.3%	42.1%	40.0%
Other	16.1%	5.3%	30.0%
Education			
High school	45.2%	42.1%	26.7%
Some college/university	41.9%	31.6%	56.7%
College/university degree	12.9%	26.3%	27.8%
Employment*			
Employed part-time	35.5%	42.1%	56.7%
Employed full-time	29.0%	15.8%	0.0%
Student	90.0%	57.9%	100%

*Participants could select more than one option

With respect to gambling severity (as measured by total PGSI scores), poker players (*M* = 2.3, *SD* = 2.3, range of 0 to 8) and VLT gamblers (*M* = 3.4, *SD* = 3.7, range of 0 to 11) did not significantly differ, *t*(48) = 1.33, *p* = .19. Of the 50 gamblers in the sample, ten met the criteria for disordered gambling (i.e., a score of 5 or greater on the PGSI). Poker players gambled an average $67.67 (*SD* = 70.60) during individual poker sessions; VLT players gambled an average of $37.89 (*SD* = 35.72) during VLT sessions. Poker players and VLT players did not differ in their total number of gambling activities during the past 12 months (*M* = 6.2, *SD* = 2.8 vs. *M* = 6.7, *SD* = 3.8), *t*(48) = 0.55, *p* = .59.

The three groups were also compared on their total scores on the Alcohol Use Disorders Identification Test (AUDIT) [40] and the Drug Abuse Screening Test (DAST-10) [41] using an ANOVA. A significant difference was found for total AUDIT scores, *F*(2, 69) = 10.92, *p* < .01, partial η^2^ = .24. Pairwise comparisons indicated that the non-gamblers (*M* = 3.0, *SD* = 2.5) had lower scores than both poker players (*M* = 8.3, *SD* = 4.8), *p* < .01, and VLT players (*M* = 8.3, *SD* = 5.7), *p* < .01. The difference between the two gambling groups was not significant (*p* = .99). Similarly, a significant difference was found for DAST scores, *F* (2, 77) = 4.67, *p* = .01, partial η^2^ = .11, with non-gamblers (*M* = 0.6, *SD* = 2.0) having lower scores than both poker players (*M* = 2.0, *SD* = 2.3), *p* = .02, and VLT players (*M* = 2.5, *SD* = 2.9), *p* = .01, who did not differ (*p* = .52).

### First fixations

The initial orienting of attention to the different images was measured by the number of first fixations to each image category, which were converted to proportions for the analyses. The proportion of first fixations was approximately equal for the four image categories: board games (23.2%), bingo (23.7%), poker (27.5%), and VLTs/slots (25.6%). To assess whether significant differences existed between the groups, the data were analyzed using a mixed-model. There was no significant main effect of Image Type, *F*(3, 231) = 2.37, *p* = .07, or Group, *F*(2, 77) = 1.26, *p* = .29. The interaction between Group and Image Type was not significant, *F*(6, 231) = 1.44, *p* = .20.

### Time of first fixation

**Fig 2:**
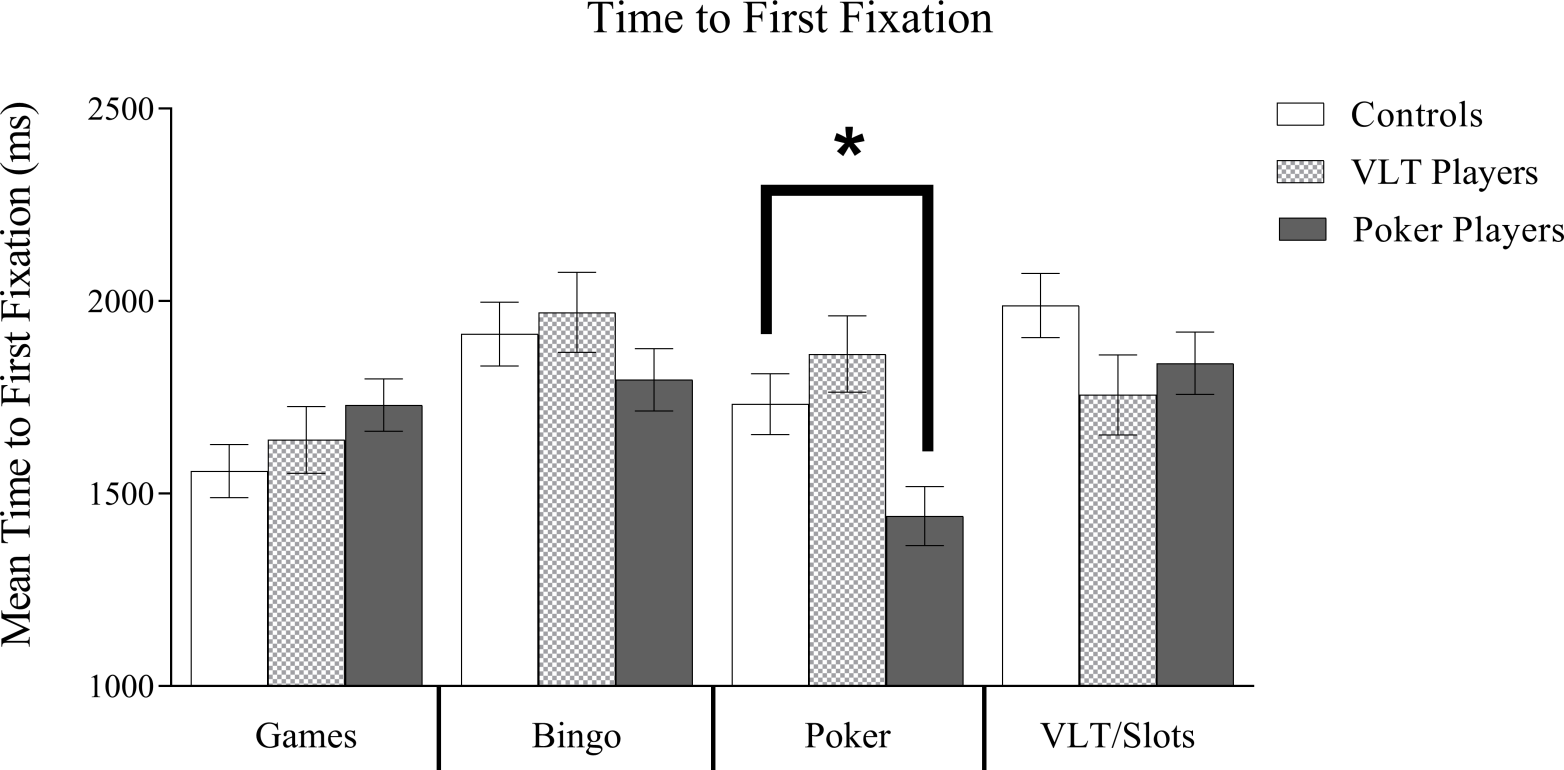
First fixation mean times. Estimated marginal means and standard errors (error bars represent SE) for Mean Time to First Fixation. There was a significant main effect for Image Type. Board games were attended to faster than bingo. The interaction of Gambling Group X Image Type was significant. The interaction revealed that poker players attended significantly faster to poker images than VLT/slots players or non-gambling controls. *p < .05.

The time of first fixation data is shown in Fig 2. The mixed-model ANOVA revealed a significant main effect for Image Type, *F*(3, 231) = 9.09, *p* < .01, partial η^2^ = .11. For the sample as a whole, pairwise comparisons revealed significantly earlier first fixations on board game images (*M* = 1642 ms, *SE* = 43) compared to bingo images (*M* = 1893 ms, *SE* = 52), *p* < .01, and VLT/slots images (*M* = 1862 ms, *SE* = 52), *p* < .01, There was no difference between board games images and poker images. *p* = .53, or between bingo images and VLT/slots images, *p* = .58. The main effect of Group was not significant, *F*(2, 77) = 1.16, *p* = .32. More importantly, there was a significant interaction between Group and Image Type, *F*(6, 231) = 3.97, *p* < .01, partial η^2^ = .09. Pairwise comparisons revealed that poker players fixated on poker images significantly earlier in the 8-second presentations than VLT players, *p* < .01, and controls, *p* = .01. In contrast, VLT players did not attend to VLT/slots images earlier than the other image types (all *p*s > .05).

### First dwell time

**Fig 3:**
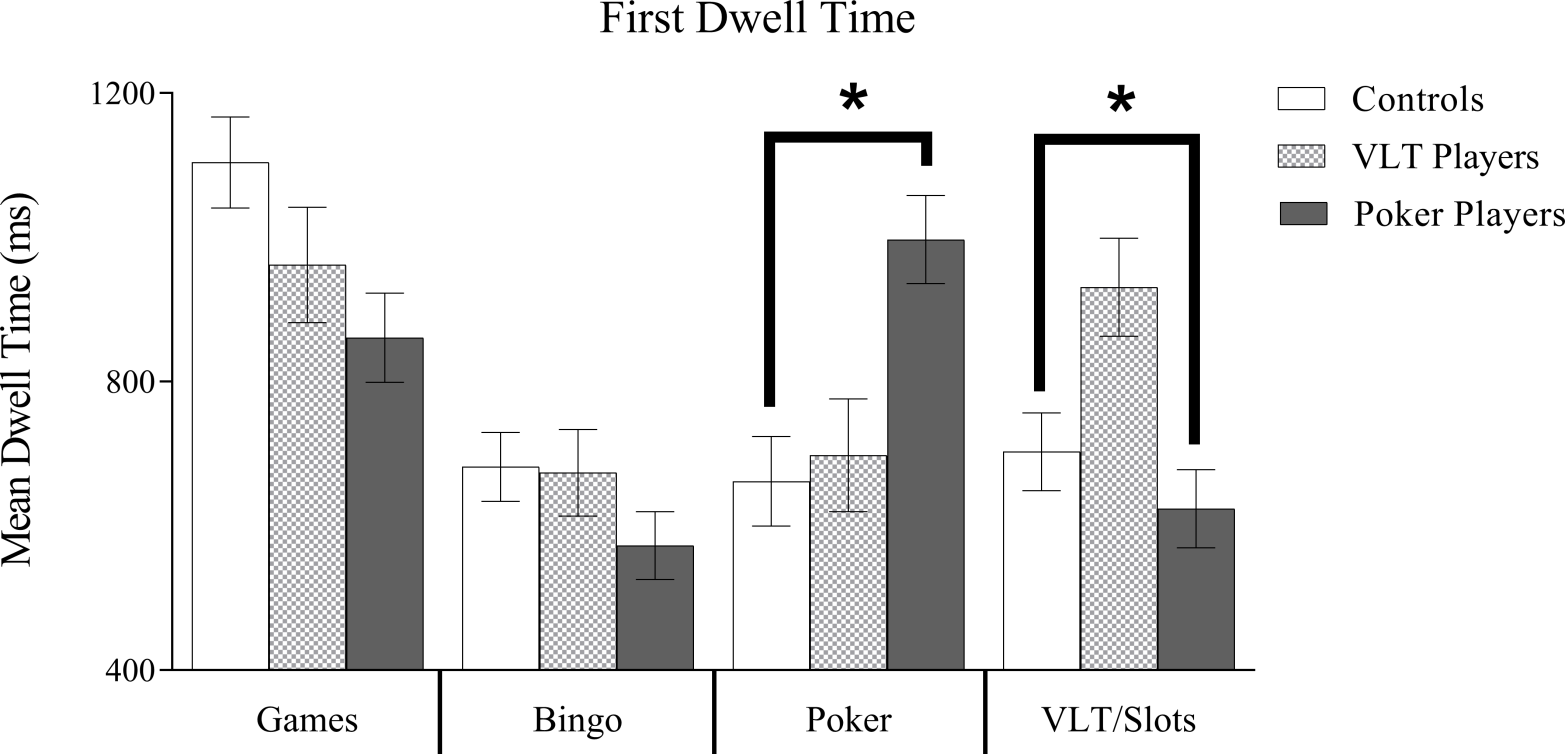
Dwell time means. Estimated marginal means and standard errors (error bars represent SE) for First Dwell Time. There was a significant main effect for Image Type. Board games received the longest dwell time and bingo the least. The interaction of Gambling Group X Image Type was also significant. The interaction revealed that poker players attended significantly longer to poker images as well as board games and VLT/slots players attended longer to VLT/slots images and board games than bingo or poker. *p < .05.

Fig 3 shows the first dwell time data. There was a significant main effect of Image Type, *F*(3, 231) = 22.83, *p* < .001, partial η^2^ = .23. The longest first dwell times were for board game images (*M* = 975 ms, *SE* = 40) and the shortest for bingo images (*M* = 643 ms, *SE* = 30), with intermediate first dwell times for poker images (*M* = 785 ms, *SE* = 39) and VLT/slots images (*M* = 752 ms, *SE* = 34). Pairwise comparisons showed significant differences between each image type (all *p*s < .01), with the exception of the difference between poker and VLT/slots images (*p* = .43). The main effect of Group was not significant, *F*(2, 77) = 0.33, *p* = .72. There was also a significant interaction between Group and Image Type, *F*(6, 231) = 10.68, *p* < .001, partial η^2^ = .22. Pairwise comparisons indicated that pokers players had significantly longer first dwell times for poker images than VLT players and controls (both *p*s < .01). Similarly, for VLT players, the comparisons revealed significantly longer first dwell times for VLT/slots images compared to poker players and controls (all *p*s < .01). Control participants had significantly longer first dwell times for board game images when compared to poker players (*p* = .01) but not VLT players (*p* = .32). There were no group differences for bingo images (all *p*s > .05).

### Total fixation time

**Fig 4:**
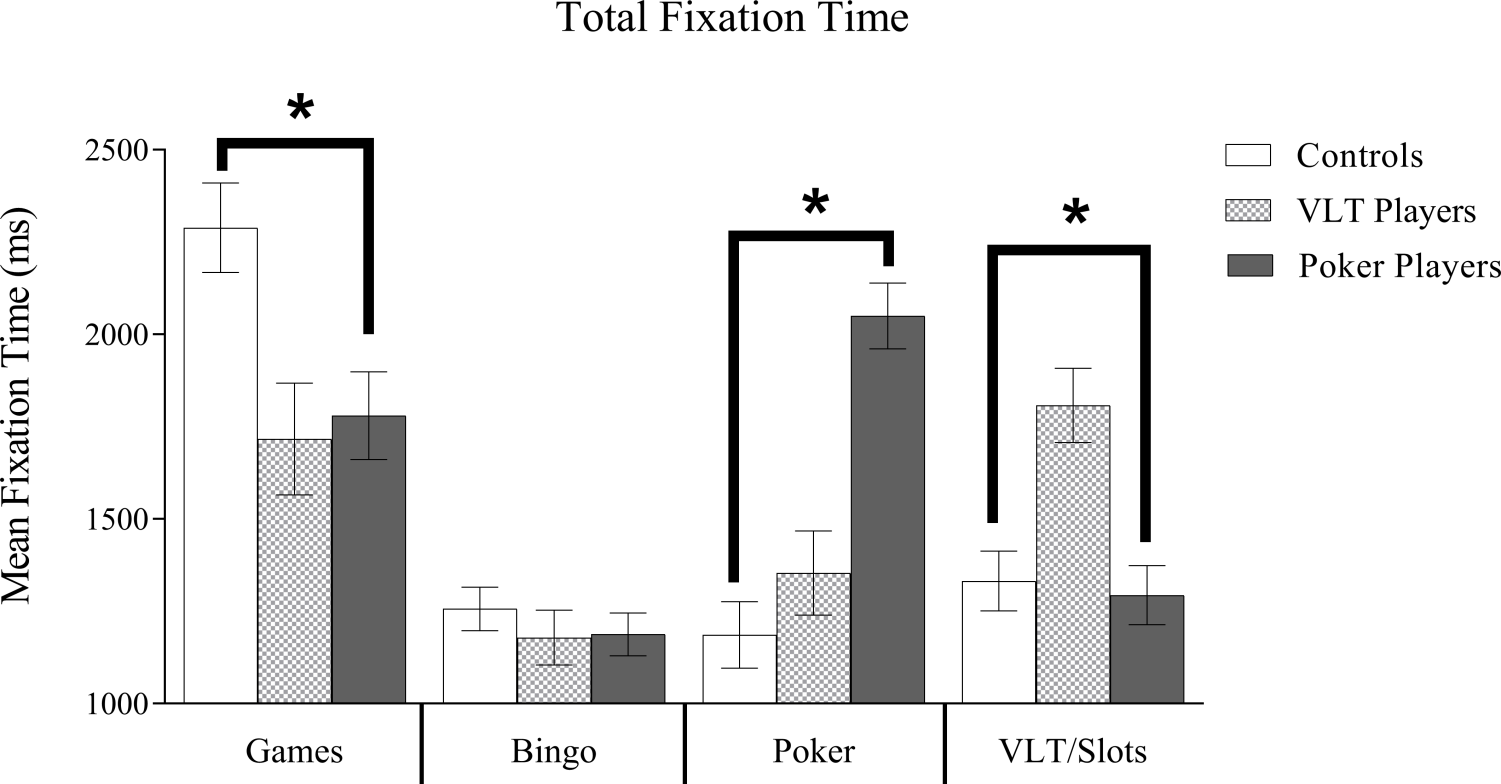
Total fixation time means. Estimated marginal means and standard errors (error bars represent SE) for Total Fixation Time. There was a significant main effect for Image Type. Board games received the greatest amount of average fixation time and bingo images the least. The interaction of Gambling Group X Image Type was significant. Poker players attended significantly longer to poker images and board games. VLT/slots players attended longer to VLT/slots images and board games while controls attended longer to board games over all other images. *p < .05.

The total fixation time data is shown in Fig 4. There was a significant main effect of Image Type, *F*(3, 231) = 23.72, *p* < .001, partial η^2^ = .24. The longest total fixation times were to board games images (*M* = 1929 ms, *SE* = 76), followed by poker (*M* = 1529 ms, *SE* = 57), VLT/slots (*M* = 1478 ms, *SE* = 51), and bingo images (*M* = 1207 ms, *SE* = 37). Total fixation times for board game images were significantly longer than for bingo, poker, and VLT images (all *p*s < .05). Fixation times for bingo images were significantly shorter than fixation times for poker and VLT/slots images (both *p*s < .05), whereas there was no difference between poker images and VLT/slots images (*p* = .50). The main effect of Group was not significant, *F*(2, 77) = 1.15, *p* = .32. Most important was the interaction between Group and Image Type, *F*(6, 231) = 12.44, *p* < .001, partial η^2^ = .24. Pairwise comparisons revealed that poker players had significantly longer total fixation times to poker images compared to VLT players and controls (both *p*s < .01). Similarly, VLT players had significantly longer total fixation times to VLT/slots images than poker players and controls (all *p*s < .01). Control participants had significantly longer total fixation times for board game images than poker players and VLT players (all *p*s < .01). Total fixation times for bingo images did not differ among the three groups (all *p*s > .10).

### Correlations between attentional bias and gambling severity

Finally, we assessed the extent to which attentional bias for gambling stimuli was associated with gambling severity scores on the PGSI. Based on the results described above, we predicted that gambling severity, as measured by the PGSI, would be positively correlated with attentional bias for images related to the preferred type of gambling (poker images for poker players and VLT/slots images for VLT players). Accordingly, two sets of correlations were conducted, the first with poker players and the second with VLT players. Total fixation time was used as the measure of attentional bias. For VLT players, there was a positive correlation between PGSI gambling severity and attentional bias for poker images (*r* = .51, *p* = .02) and for VLT images (*r* = .47, *p* = .04). For board game and bingo images, the correlations were not statistically significant (*r* = -.24, *p* = .33 and *r* = -.11, *p* = .67, respectively). For pokers players, the correlation between gambling severity and attentional bias for poker images was not statistically significant (*p* = .17). Lastly, for board game (*r* = -.15, *p* = .42), VLT/slots (*r* = -.15, *p* = .43), and bingo images (*r* = -.15, *p* = .42) the correlations were not statistically significant.

## Discussion

The purpose of this study was to compare, for the first time, attentional biases for preferred gambling activity between non-strategic gamblers (VLT players) and strategic gamblers (poker players). A control group of non-gamblers and two types of alternative stimuli (i.e., board games, bingo) were included to more definitively establish the specificity of gambling-related attentional biases. Eye movements of participants were recorded while they viewed gambling and non-gambling related images throughout an 8-second presentation. Attention to the images was measured via four dependent variables recorded automatically by the eye-tracking system: first fixations, time of first fixation, first dwell time, and total fixation time. Based on previous literature and theory, it was predicted that gamblers would exhibit an attentional bias for their preferred form of gambling. Control participants were not expected to preferentially fixate on any specific image category; they were expected to attend to the images equally. The analyses supported most of these predictions.

First, poker players exhibited a strong attentional preference for poker-related imagery. These individuals were more likely to initially fixate on poker images, focus on poker images significantly earlier than other images, and preferentially attend to poker images for significantly longer periods of time than VLT players or control participants. For VLT players, the results also supported the prediction of attentional bias specificity. Fixation times on VLT/slots images were longer than for other stimuli, and VLT players had significantly longer first dwell times for VLT/slots imagery and longer total fixation times to VLT/slots. Interestingly, the prediction that non-gamblers would attend equally to all the images was not supported, as control participants instead preferentially attended to board game images. One explanation for this finding is that the non-gamblers were disinterested in gambling imagery and instead attended to the only non-gambling images in the displays (the board game images). Non-gamblers often cite dislike of gambling as a reason for avoiding gambling in general [42], and if true for the non-gamblers in our study this would be consistent with their disinterest in gambling-related images. Another possibility is that because the board game images were more varied than the gambling images (which consisted mainly of images of cards, slot machines, and bingo), their relative novelty may have attracted the attention of participants and this difference may have contributed to the differences in fixation times for board game and gambling images. Many of the board game images depicted well known commercially popular games (e.g., Trouble, Scrabble, Monopoly). It is possible that personal history with these games may have unduly influenced attention toward them.

Taken together, these findings provide further evidence for the existence of an attentional bias toward gambling stimuli in a sample of regular gamblers. Equally important, our predictions regarding the heterogeneity of gambling and the specificity of attentional biases according to preferred gambling activity were supported. Crucially, few (if any) studies on attentional biases have taken gambling activity preferences into account. For example, as noted, while both Brevers et al. [25] and Grant and Bowling [26] used a wide array of gambling images in their studies, their stimuli were not matched with their participant’s gambling habits. The results of our study indicate that the match between a participant’s preferred form of gambling and the gambling imagery used to measure attentional bias is a crucial consideration. Our findings further suggest that considerable direct experience with a specific gambling activity (poker, VLT playing) may be required for an attentional bias to develop. As such, imagery belonging to any ‘gambling’ category may be insufficient to illicit a strong attentional response in a gambler who has little experience with other types of gambling. In this respect, our findings provide support for Robinson and Berridge’s [7] theoretical perspective that attentional biases likely develop through associative learning mechanisms as a consequence of repeated experience with an activity. Our results indicate that the degree of alignment between gambling stimuli and the participant’s gambling history should be carefully considered in experimental designs.

Our findings could have important clinical implications for the treatment of gambling disorder. In particular, there has been growing interest and effort directed toward the development of effective and easily implemented attentional bias modification programs [43–44]. These programs are designed to teach individuals who are experiencing an attentional bias toward addiction-related cues to divert their attention to more neutral stimuli via a visual probe procedure. This is done over a series of repeated administrations, with the end goal of complete retraining of attentional processes [44]. While most attentional retraining programs have been designed for substance users, findings from neuroimaging studies suggest that similar modification programs could also benefit disordered gamblers [45]. In fact, an ongoing randomized clinical controlled trial in the Netherlands is currently testing the effectiveness of both attentional and approach bias training (vs. placebo versions of both programs) on future gambling behavior in a sample of disordered gamblers [46]. The results of our study could influence the design of these types of programs. In particular, our findings highlight the importance of personally-relevant gambling cues over non-gambling and ‘other’ gambling stimuli. Future studies directly testing this possibility are warranted.

In addition to the potential clinical implications of our results, our study also illustrates the advantages of eye-gaze tracking as a means for measuring attention in gambling research. Unlike other attention tasks (e.g., the Stroop task, dot-probe tasks), tracking eye movements allows for a moment-to-moment assessment of attention while participants view a display of competing gambling imagery. The issue of assessing individual variability as well as fluctuations in attention during attentional bias tasks has been recently highlighted as an important research direction in the alcohol literature (e.g., [47–48]). In our study, eye-gaze tracking permitted a nuanced comparison of attentional bias for specific types of gambling in real-time.

There were several limitations of this study that should be considered. First, given that only males between the ages of 18 and 35 years old were recruited, the sample was not representative of the broader population of gamblers. Second, the mean PGSI score for the gambling groups was fairly low, which limits the extent to which we can generalize our results to clinical samples of disordered gamblers. Third, as noted, the novelty of the board game images may have had a large influence on the control participants’ attention. Future studies may wish to use control images that are more closely matched to gambling images in their degree of diversity. Finally, ratings of subjective cravings for gambling were not measured. Ratings of craving prior to and following cue exposure would permit mediation analyses and could shed light on possible underlying mechanisms of gambling-related attentional bias (e.g., the link between cravings and attentional biases for gambling-related imagery).

## Conclusions

In conclusion, the present study is the first attempt to assess the specificity of gambling-related attentional biases according to preferred gambling activity. As predicted, greater attention toward poker imagery was observed in poker players and greater attention toward VLT imagery was observed in VLT players. These findings provide support for the specificity of attentional biases toward stimuli that are congruent with gambling histories. Our study also highlights the advantages of using eye-gaze tracking to measure attentional biases in gambling. Although widely employed in substance use studies, there are comparatively few eye-gaze tracking studies in the gambling literature. Further research could explore the utility of fixation measures to identify attentional processes underlying gambling behavior (e.g., patterns in the sequence with which gambling and non-gambling images are attended in experimental displays).

## Supporting information

S1 File Attentional biases dataset_2017. SPSS dataset containing the raw and anonymized data of all 80 male participants (31 poker players, 19 VLT/slots players, and 30 non-gambling controls).
(SAV)
